# Discharge processes and medicines communication from the patient perspective: A qualitative study at an internal medicines ward in Norway

**DOI:** 10.1111/hex.13232

**Published:** 2021-03-24

**Authors:** Stine Eidhammer Rognan, Sofia Kälvemark Sporrong, Kajsa Bengtsson, Helene Berg Lie, Yvonne Andersson, Morten Mowé, Liv Mathiesen

**Affiliations:** ^1^ Department of Pharmaceutical Services Oslo Hospital Pharmacy Oslo Norway; ^2^ Hospital Pharmacies Enterprise South Eastern Norway Oslo Norway; ^3^ Department of Pharmacy University of Copenhagen Copenhagen Denmark; ^4^ Department of Pharmacy University of Oslo Oslo Norway; ^5^ Division of Medicine Oslo University Hospital Oslo Norway; ^6^ Institute of Clinical Medicine University of Oslo Oslo Norway

**Keywords:** hospital discharge, medicines communication, observational study, patient empowerment, patient perspective, patient‐centred care

## Abstract

**Background:**

Patients are expected to participate in the hospital discharge process, assume self‐management after discharge and communicate relevant information to their general practitioner; however, patients report that they are not being sufficiently empowered to take on these responsibilities. The aim of this study was to explore and understand the discharge process with a focus on medicines communication, from the patient perspective.

**Methods:**

Patients were included at a hospital ward, observed during health‐care personnel encounters on the day of discharge and interviewed 1‐2 weeks after discharge. A process analysis was performed, and a content analysis combined data from observations and data from patient interviews focusing on medicines communication in the discharge process.

**Results:**

A total of 9 patients were observed on the day of discharge, equalling 67.5 hours of observations. The analysis resulted in the following themes: (a) the observed discharge process; (b) patient initiatives; and (c) the patient role. The medicines communication in the discharge process appeared unstructured. Various patient preferences and needs were revealed. The elements of the best practice structured discharge conversation were observed; however, some patients did not have a discharge conversation at all.

**Conclusions:**

The study contributes to a broader understanding of the discharge process, how patients experience it, including their role. It is evident that the discharge process is not always tailored to meet the patients’ needs. More focus on early patient involvement and communication, in order to better prepare patients for self‐management of their medications, is important for their health outcomes.

## 1 INTRODUCTION

Patients are expected to participate in the hospital discharge process, assume self‐management after discharge and communicate relevant information to their general practitioner; however, patients report that they are not being sufficiently empowered to take on these responsibilities.^1^, ^2^, ^3^ Changes made in the medicines treatment without ensuring the patient's motivation and skills can give rise to misunderstandings.^4^, ^5^ Elderly patients may be particularly vulnerable, by struggling with unfamiliar vocabulary or suffering from hearing difficulties or cognitive impairment.^5^, ^6^, ^7^, ^8^, ^9^, ^10^, ^11^

Previous studies have identified variation in the duration of the discharge process (from a few hours to a few days). Furthermore, the time of day when patients were determined to be ‘medically fit’ for discharge affected HCPs’ level of stress, especially at the end of shifts when they had to make room for new patients.^2^, ^11^, ^12^, ^13^ The HCPs’ time efficiency on the day of discharge seen from a patient perspective may be incomprehensible and can affect the patients’ dignity.^14^

The patient's right to health and medicines information is enshrined in Norwegian legislations.^15^, ^16^ The information should be adapted to the individual, and HCPs should ascertain the patient's understanding.^16^ In a national patient safety campaign, safe hospital discharge was a target area, with ‘structured discharge conversation’ as one of the specific actions and with the intention that the patient and/or next of kin exchange information and clarify any uncertainties with the HCPs.^17^

The hospital discharge process has to a substantial degree been viewed through other glasses than the patient's, even though the patient experience is central to high‐quality care.^3^ It is challenging to develop a systematic approach to translating patient experience into customized solutions, because of the conflicting goals such as patient‐centred care vs. organizational health system demands.^1^, ^10^, ^14^ To make discharge processes efficient, patient values, perceptions, experiences and knowledge must be appraised.^1^, ^7^, ^13^, ^18^, ^19^ In this substudy, we explore the discharge process with a focus on medicines communication from a patient perspective.

## 2 METHODOLOGY

This is a qualitative study, consisting of primarily unstructured observations, semi‐structured interviews and medicines reconciliations. The results presented are part of a larger study, observing the patients for a longer part of their hospitalization. The aim of the main study was to explore and understand the patient perspective of medicines communication during hospitalization and the discharge process.

### 2.1 Setting and sampling strategy

Patients were included and observed at an internal medicines ward at a university hospital in Norway. The interviews were performed 1‐2 weeks after discharge in the patient homes, at a short‐term nursing home, at a café or by telephone.

Patients were included from September to December 2019, close to the day of their planned discharge. Thereafter, the patients were followed during HCP encounters through to hospital discharge.

The sampling method was purposive; to ensure heterogeneity, the observers (KB, HBL and SER) selected eligible patients based on sociodemographics (eg gender, age, education and ethnicity), diagnoses and assumed length of hospital stay. Eligible patients should be ≥ 18 years old, home‐dwelling, responsible for their medicines administration prior to hospital admission and expected to be discharged to their homes or a short‐term nursing home department. Pre‐terminal or cognitively impaired patients were not eligible.

### 2.2 Data collection

The observers (pharmacy students or pharmacist, authors KB, HBL and SER) got relevant training through performing a pilot study. In the pilot study, authors KB, HBL, SER and LM observed together and then discussed any differences in observations. During the observations, the observer was present and identifiable, but without any role in the social setting.^20^

In Norway, HCPs at hospitals normally wear white uniforms. The observers disclosed their background, but dressed to appear more as ‘the girl from university’ than HCPs.^21^, ^22^ The observers wore a yellow T‐shirt with the word ‘observer’ across the front. Observations took place Monday to Friday (and occasionally in weekends) from 8:00 to 15.30, covering the period when most hospital activities normally take place.

Relevant information from the observations was documented in a form, developed and tested in the pilot study (see Appendix A). The focus of the observations was medicines communication, that is content and contextual factors, activities and interactions. All encounters with physicians or nursing staff that could potentially include medicines communication were observed. All patients were mainly observed by one observer. A second observer performed observations if necessary, for example if the first observer needed a (lunch) break. Having a main observer maintained continuity, and adding a second enhanced consensus discussions. The observations were audio‐recorded if the patient stayed in a single room and if both the patient and HCPs consented.

The interviews were conducted by KB and HBL and were audio‐recorded if the patients consented. At the interviews, an interview guide (Appendix B) comprising a list of items and probing questions guided the interviewer. The focus of the interviews was the discharge process and important factors related to the medicines treatment from the patient's perspective. In conjunction with the interview, a medicines reconciliation was conducted according to the integrated medicines management (IMM) model adapted to the Norwegian setting.^23^

### 2.3 Ethical considerations

Written informed consent was obtained from patients and HCPs prior to the observations. Patients gave an additional informed consent to the interview. Data were de‐identified and stored in a protected area at the university. The Regional Ethics Committee assessed the study and found no ethical approval necessary. The study was approved by the Privacy Ombudsman and the Hospital Investigational Review Board (08 March 2019, reference number 2019/6465). A gift (value of 150 NOK—13 € or 14 USD) was given to the patients at the interviews.

### 2.4 Analysis

We analysed the part of the data related to the discharge process, covering the day of discharge. Criteria for inclusion into the analysis were as follows: (a) existing data from observations on the day of discharge, and (b) interview after discharge.

Data were transcribed consecutively to prevent memory bias. The first codes were inductively derived from the first three transcripts (covering all observed encounters for each patient), individually and in several consensus sessions (by KB, HBL, SER, SKS, YA, and LM). This resulted in a preliminary codebook with codes relevant to the overall research question.^24^ The coding of one interview transcript with a fourth patient was discussed in an additional consensus session (by KB, HBL, SER, SKS, YA), and the codes slightly changed. All transcripts were then coded using NVivo qualitative analysis software ^25^ (by KB, HBL, SER); during this part of the process, new codes were added to the codebook. A last revision of the coding was made using the final codebook. All coding made by one person was audited by the others. Code groups were then condensed into units of meaning with a focus on medicines communication in the discharge process. Furthermore, after searching for similarities, differences and connections cross‐case, the results were clustered into themes.^26^

To map the process on the day of discharge, the original transcripts were used in an additional analysis in order to capture the sequence of events.

Through constantly comparing experiences and responses of the participants during the sampling, we appraised the richness and depth of the data. After 15 observed patients, we concluded we had reached saturation.^27^ Of the 15 patients, 6 were excluded for this analysis as they lacked the interview (n = 2), observations on the day of discharge (n = 1) or both (n = 3). Of patients approached, one declined to participate.

The patients are presented with pseudonyms. The text and quotes are from observations if not specified with interview or ‘int’.

The result section consists of the observed discharge process, and the thematic analysis of observations and interview data.

## 3 RESULTS

Nine patients were included in the analysis, eight Norwegians and one with another European citizenship. The data material consisted of 303 pages from 67.5 hours of observation on the day of discharge and 8.5 hours of patient interviews. The median length of hospital stay was five days (range 4‐18), and the patients were observed for a median of two days (range 1‐6) before discharge. The median length of the interviews was 55 minutes (range 33‐87). Demographics and other quantitative data are presented in Table 1.

**TABLE 1:** Demographics of the patients

Demographics	(n = 9)
Sex	
Male	4
Female	5
Age, median (range)	71 (49‐90)
Education	
Compulsory school/unknown	2
Upper secondary school	4
University	3
Main diagnoses according to discharge summary	
Atrial fibrillation	2
Pulmonary embolism	2
Pyelonephritis	1
Pulmonary oedema	1
Myocardial infarction	1
Gout	1
Heart failure	1
Hospital environment	
Single‐bed room	6
Multibed room (sharing with 1 or 2 other patients)	3

### 3.1 Mapping the discharge process

Every patient experienced a unique discharge process with variation in timing, duration and communication. Some patients received elements of the best practice structured discharge conversation, that is timely information about the discharge, where the HCPs took time to listen, revising the information together with the physician.

**FIGURE 1:**
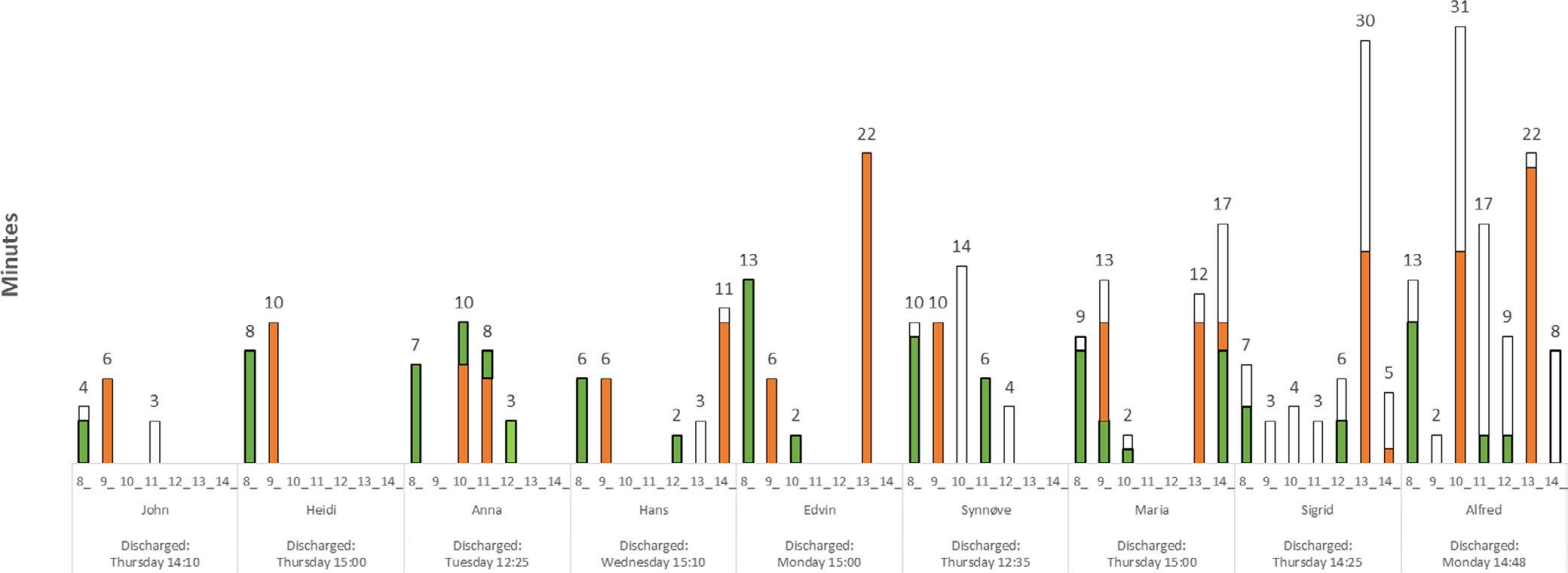
Road map of the day of discharge. The *y*‐axis represents duration of the encounters (minutes). Numbers represent total duration per hour. The *x*‐axis shows the patients and the time of the encounters with HCPs during the day (from 08:00 to 15:30). Standard measurements and administration of medicines are marked with green. Ward round and discharge encounter with doctor are marked with orange. White represents ‘other encounters with HCPs’. 'Discharged' indicates the end of the discharge process, here defined as the patients' departure from the hospital ward

The patients had a median number of 10 encounters (range 5‐23) with a median number of four different HCPs (range 2‐7) on the day of discharge (see Table 2). The median total time of encounters was 43 minutes (range 13‐102) (Figure 1).

**TABLE 2:** Characteristics of HCPs observed on the day of discharge

	n (% female)
Physicians	
Senior consultant	4 (25)
Junior physician^a^	6 (83)
LIS‐2‐3, n = 2	
LIS‐1, n = 3	
Medical student, n = 1	
Registered nurses	9 (78)
Nurse assistants	1 (100)
Nurse students	5 (100)
Pharmacy technicians	1 (100)

^a^ Intern/specialization practice. LIS, ‘lege i spesialisering’—in English, junior physician undergoing a specialization programme, consisting of levels 1‐3, which has to be completed before qualified to work as specialists or senior consultants.

The patients were discharged during regular working hours (Monday‐Friday). Little activity was observed during the weekend, there was, for example, no ward rounds. Generally, the patients experienced a lot of waiting during the hospital stay. They did not seem to experience the day of discharge as noticeably busier than other days; four of the patients had several days with 15‐24 minutes longer duration of encounters with HCPs than at the discharge day.

Almost all patients experienced some main encounters, generally following the same scheme and often involving medicines communication. The main encounters consisted of standard measurements (eg blood pressure and temperature), morning medicines administration, ward round and discharge conversation (Table 3). Some patients had more than one 'other encounters'. Most often, the ‘other encounters’ concerned activities such as administering more medicines or patient call for HCPs; however, sometimes they may have appeared as excessive from the patient perspective, for example 13 minutes with a pharmacy technician explaining about medicines administration, although the patient had been told that the home nurse services would be responsible for the medicines administration post‐discharge. Importantly, the pharmacy technician had not been informed about this.

**TABLE 3:** Overview of encounters including duration in minutes on the day of discharge from hospital

Encounters (n = number of patients)	Median duration in minutes (range)
Main encounters	
Standard measurements (n = 8)	4.25 (2‐12)
Morning medicines administration (n = 9)	3 (1‐8)
Ward round (n = 9)	10 (6‐17)
Discharge conversation (n = 6)	8 (1‐21)
Other encounters	
Medicines administration, in addition to morning medicines (n = 5)	2 (1‐4)
Coordination of discharge (n = 4) (Transportation, communication with municipalities about post‐discharge situation, dispensing^a^ medicines or medical equipment)	3 (1‐13)
Other measurements (n = 4) (Blood sugar, weight, orthostatic test, extra measurements)	5 (1‐12)
Meals served (n = 4)	2.5 (1‐3)
Patient call for HCPs (n = 3)	3 (1‐8)
HCPs checking whether everything was ok (n = 2)	2 (1‐3)
Removal, control or application of medical equipment (n = 2)	10.4 (7‐14)
Nutritional advice (n = 1)	4 (4)

^a^ Dispensing**:** delivery of medicines at the hospital ward intending to prevent interruptions in medical treatment post‐discharge.

Tentative discharge dates were often postponed multiple times. The patients often got the first clue about the current status of discharge during morning medicines administration; however, the final decision came during ward round (typically 09:15‐10:15 in the morning) or sometimes later if pending on biochemical status.

*Doctor*: If we say you stay until tomorrow, are you okay with that? I expected to be able to go home to my husband the first night. *Synnøve (♀, 84, int)*

Seven patients had a discharge conversation; the median duration was 8 minutes (range 1‐21). One discharge conversation was not observed, as it took place in the common area at the ward, and is not included in the calculation of the median. Two patients had discharge conversations shorter than 3 minutes. These patients had informative ward rounds (15 and 17 minutes) just before the discharge encounter. In the two longest discharge conversations (14 and 21 minutes), a junior physician went through a customized discharge summary with the patients, who also had many questions and comments. The content of the physicians’ information in these encounters differed from the four shorter discharge conversations. In the shorter conversations, the physician more briefly described the content of the discharge summary and then directed the patients to the discharge letter if they, their next of kin or home nurse services should have any questions. Next of kin were not present in any encounters on the day of discharge. However, a physician had been in telephone contact with the next of kin of one of the elderly patients.

Two patients did not have any discharge conversation. Both of them experienced unclear information during the ward round as to whether there was going to be a discharge conversation or not. Regarding the first patient, the physician had delivered the discharge summary and described the content on the previous day. For the second patient, the ward round lasted for ten minutes, and it was unclear whether the doctor was coming back for a discharge conversation. The patient ended up having to, on her own initiative, collect the discharge summary from the nurse. The content of the discharge summary was not described to her.

The information around discharge was a bit lacking, it was just ‘yes you can go’. *John (♂, 58, int)*

For some patients, a multibed room meant lack of confidentiality, because other patients were present.

Lying in a triple room is the same as having a discharge conversation in the common areas. I do not think it is okay when sensitive information can be overheard by other patients. *Heidi (♀, 53, int)*

The discharge encounters were informative with a retrospective content. The patients often got a combination of written and oral information about their main cause of hospitalization, treatment and recommended follow‐up. Sometimes, they were also informed about when and why they should use their medicines, the duration of treatment, common side‐effects and when it was important to get in touch with HCPs, including why. Systematic communication techniques were not apparent in the discharge counselling; for example, no specific techniques to ascertain patients’ understanding were consistently used. The decisional responsibility could also be ambiguous; physicians were sometimes alternating between deciding, recommending and leaving decisions to a patient.

*Junior physician*: **You** should go to and tell **him** [specialist], what **we** [hospital doctors] have already decided /…/ If the specialist needs to investigate something else, then you [specialist and patient] can find an agreement /…/ I think it might be a little up to **him** [specialist], if **we** [hospital doctors] have already covered it /…/Does that sound okay to **you**?’

In the interviews, all patients stated that they intended to take their medicines as prescribed and that they had no problem to remember to take them. However, six out of nine patients had discrepancies between the medicines list decided upon at discharge and their current or planned use (see Table 4). Many of the discrepancies could be explained by poor or incomplete information from HCPs.

**TABLE 4:** Discrepancies between the medicines list in the discharge summary and patients’ actual use of medicines revealed during the semi‐structured interviews (including medicines reconciliation), 1‐2 weeks post‐discharge

♀/♂, age	Type of discrepancy	Description of discrepancy
♂, 79	Using a medicine not included in the discharge summary	Prednisolone [against gout]. Possible misunderstanding. Inconsistency between oral and written information
♀, 89		Zolpidem [against sleep problems]. The hospital prescribed zopiclone [against sleep problems], which the patient used in addition to zolpidem
♂, 58	Intentionally not using the medicine as described in the discharge summary	Salbutamol [against asthma]. The patient was using this on demand, and not regularly as stated in the discharge summary
♀, 83		Amitriptyline [against depression or pain]. The patient told HCPs that she did not use this medicine
♀, 71		Vitamin B supplement, which was not dispensed with the multidose‐dispensed medicines
♂, 61	Unintentionally planning to discontinue medicines	The patient had the perception he was supposed to discontinue the five newly started medicines [against high blood pressure, atherosclerosis, high cholesterol and enlarged prostate] after emptying the packages containing 98 tablets

### 3.2 Themes emerging from observations and semi‐structured interviews

The thematic analysis of observations and interview data resulted in two themes: (a) Patient initiatives and (b) Patient role. The themes describe the patients’ personal preferences, needs and personality.

#### 3.2.1 Patient initiatives

On the day of discharge, the patients generally expressed basic needs; they wanted to take a shower and dress properly. In general, the patients were active. To differing degrees, they asked questions and commented on the process, for example what is going to happen, when is the doctor's visit and when can I go home. The nurse assistants often had to forward the question to a nurse, and the nurse often had to forward it to a doctor.

Well, I suppose it was up to me to ask about it. I had the opportunity. *Sigrid (♀, 71, int)*

The patients asked questions about the medical treatment. They seemed to prefer to keep some control of which medicines and dosages that were administered, or in some cases critically address treatment choices and make alternative suggestions. Even patients not responsible for handling their medicines after discharge asked questions and expressed a need to remain in some sense of control.

Do I have to use lisinopril [against heart failure]? The GP told me I didn’t have to because I got so dizzy. Is it because of the heart? (…)

I’m also a little concerned about allopurinol [against gout], why are you starting it now? I’m not sure if my kidneys can tolerate it. *Alfred (♂, 80)*

Then I have some questions for you. I wonder about that non‐prescription medicine, I have heard it is difficult to get a hold of. How can we get it fixed? It could be a good idea to have it at hand, just in case. *Edvin (♂, 61)*

Some patients expressed high confidence in their own knowledge regarding medicines, asking questions to the HCPs to ‘make them think, because they were the ones with the knowledge’, to strengthen their own knowledge or to make sure that HCPs did their jobs properly.

I knew which medicines I used. I was very careful at the hospital. I checked if they had actually remembered and that it was done right.

*Heidi (♀, 53, int)*

The patients seemed to want to prepare themselves for self‐management after going home and to check their own understanding prior to discharge. Some patients asked about prognosis and about necessary precautions, for example what they should do or not do if they did not feel well. They also wanted to have information clarified or repeated if they did not understand or if they could not recall the information they had been provided with, for example dosing or duration of treatment.

Will I still experience pain? *Anna (♀, 90)*

I have used some of that drug [glyceryl trinitrate, against angina] preventively and it has helped. Can I continue with that? I have read that you should sit down while taking it, if not you will feel dizzy. If I'm on my way to the store, I can't sit down on a rock along the way? I don’t want to use a walker! *Synnøve (♀, 84)*

The patients seemed to want to make sure that everything necessary was prepared before they left the hospital. For example, they expressed their expectations regarding prescriptions and dispensing to prevent interruptions in medical treatment and further follow‐up. One patient was offered medicines delivered to bedside, but because he had to manage on his own from now on, he went to the pharmacy by himself.

These tablets, are they something I get here? *Edvin (♂, 61)*

I thought about emh … medicine, can I get it through the pharmacy? Can they deliver it to me while I’m here? *Sigrid (♀, 71)*

Everything is new to me, it's a bit of a buzz. If you could fill the pill‐dispenser until Thursday, the home nurse services will take care of it after that. Will you notify the pharmacy? Will the GP and the specialist get the same papers? What do I need to keep an eye on? *Anna (♀, 90)*

All but one patient were eager to be discharged. This patient expressed being uncomfortable, not feeling well nor safe, and extra reassurance from the HCPs was needed. Another patient experienced symptoms on the day of discharge, but considered to keep this information a secret in fear of delayed discharge. Ultimately, the patient decided to disclose the symptoms; hence, the nurse could inform the doctor who could then re‐evaluate the dosage before the patient was discharged.

You may want to postpone another day if potassium is still low? *Maria (♀, 49)*

How do I notice if the dosage of the new medicine is too high? I was a bit shaky earlier today, but I didn't dare say it because I was afraid the doctor would change her mind and I had to stay another day. *Synnøve (♀, 84)*

#### 3.2.2 The patient role

The patients did not always ask questions to HCPs even if they had the opportunity and had questions. Some patients had a background from the health‐care system, or they had close relatives with such backgrounds with whom they conferred. Some, both younger and elderly with help from next of kin, claimed to read results from Internet searches critically, for example by using patient information leaflets or the health authorities' web pages. Others did not seem to be source‐critical at all. Some patients allegedly searched for information to check whether the HCPs were doing their job.

I googled the minute I got new medicines at the hospital to check what side effects to expect, and to check if I was right about the administration. *Heidi (♀, 53, int)*

The doctors will hate this answer; I just went on Google to see what the drug was, to check side effects and against other medicines. You just have to read the results with a reasonable frame of mind. *John (♂, 58, int)*

Then I go online. And navigate to… A medical, what is it called. My daughter helps me, she has an overview. I have always known where to find data on medications. *Sigrid (♀, 71, int)*

Some patients did not want to, or did not think they had sufficient competence to, discuss medicines or to be involved in the decisions related to medicines.

I will not say that they informed me about the drugs, but I got the ones I was supposed to have at certain times and I just took them and I thought that it is how it is supposed to be, there wasn't any questions about that… *Hans (♂, 90, int)*

I think … in order to discuss drugs, I have to be a doctor myself. I can't decide anything without a doctor, when they say this is for me, if it helps me, then I have to trust it. *Maria (♀, 49, int)*

All patients showed willingness to take on responsibility and to make sure that necessary follow‐up appointments with their GP were made. One patient contacted the home nurse to arrange a home visit, and another wanted to stay some days at a short‐term nursing home department before going home. Post‐discharge, at the time of the interviews, some patients still seemed to struggle to cope with their altered physiological state. Others seemed to have more or less adapted, with a satisfactory level of self‐management achieved. Some patients had unanswered questions, for example about possible side‐effects and whether the duration of the treatment was ‘time‐limited or for lifetime’.

I checked a lot of details with the pharmacy staff afterwards. I had two questions in my head to ask the [hospital] doctor and I forgot. My first question was going to be ‘is there anything to avoid, while I’m on that medicine’ and the second question was ‘Is there a question I should have asked you that I haven’t?’ *John (♂, 58, int)*

There are 98 tablets in each package. I guess the hospital doctor thought that these medicines should be phased out gradually. *Edvin (♂, 61, int)*

One feels unsafe when it has to do with the heart. *Synnøve (♀, 84)*

## 4 DISCUSSION AND CONCLUSION

### 4.1 Discussion

We found that the discharge process, including the discharge conversation, offers little time for the patients to prepare for self‐management. Although all patients stated that they intended to adhere to the medicines treatment plan, several had a different understanding or another plan for managing their medications. Some patients experienced many encounters with different HCPs on the day of discharge, which can increase the risk of communication failures.^12^ The insufficient abilities of HCPs to prepare patients for self‐management seem to be a universal problem.^1^, ^10^, ^11^, ^28^, ^29^, ^30^, ^31^ However, discharge conversations motivating patients to seek instructions from HCPs traditionally have been, and still seem to be, key to patient participation and self‐management.^11^, ^32^, ^33^ Our study adds to the current knowledge through comprehensive observations covering all encounters, not only the discharge conversation, in combination with interviews. Thus, it provides a rich and thorough picture of patients’ journey from hospital to home—from the patients’ perspectives. We propose implementing more patient‐centred activities during the entire hospital stay to better prepare the patients for self‐management of their medications.

Because the final decision of discharge was often sudden, the patients had little time to take initiatives and ask questions. The discharge conversation was not given much time, and some patients did not have a discharge conversation at all. Although the day of discharge may be busy for the HCPs, we found that it is not necessarily characterized by ‘time pressure’ from the patient's perspective.

The discharge conversations mostly had a retrospective focus, the physician going through the medicines list at the end. Furthermore, HCP’s initiatives to facilitate patient understanding, for example using teach‐back or pausing to allow the patient to think and to clarify any misunderstandings, seemed random or related to individual HCPs’ communication styles. The discharge conversations generally appeared less structured than recommended by national policies.^17^ In summary, we identified variation in timing, duration and content of the discharge conversations. We assume that the underlying causes are complex, ranging from variation in patient and HCP personalities and knowledge to ward staffing levels. Rushed discharge conversations, lacking standardized processes for informing patients about their medicines, have also been found previously.^11^, ^28^, ^34^ Some may argue that discharge conversations are impossible to standardize, because the approach to patient involvement should be tailored.^35^ However, it can be questioned whether the current form of discharge conversations is in the patient's best interest, or whether it functions more as a checklist item for HCPs.

Most patients were proactive, able to be involved and seemed motivated to seek instructions from HCPs. Accordingly, patients seemed to want to prepare themselves for self‐management and to check their own understanding. However, it was evident that patients could have been provided with more information, regardless of the patient initiatives, to understand the discharge process, for example more information about how they could become more involved, that is what they could ask, making clear how and when there was room for them to speak up. This could improve their self‐management and post‐discharge outcomes.^1^, ^34^, ^36^ Patients value that responsibility and communication is clear, unambiguous and transparent, thus being provided with all the information necessary to self‐manage.^2^, ^7^, ^10^ The passive role of some patients in the discharge process could have been a consequence of lacking information, personal resources, capabilities, discipline, ambiguities regarding responsibilities or a combination of these factors.^2^, ^37^ Previous studies have shown that when the time for communication is limited and the location is suboptimal, patients may find it difficult to initiate communication with HCPs, and be afraid of being a bother.^10^, ^17^, ^32^, ^34^ As some patients in our study chose not to ask all questions they had, information in the end will be missing.

In the interviews, all patients stated that they intended to adhere to their medicines; however, for more than half of them, their ability to be so was challenged. Among factors associated with poor adherence, ‘health‐care–related factors’ ^30^ with poor or incomplete communication from HCPs could fully or partly explain the discrepancies between the medical treatment plan in the discharge summary and the patients’ interpretations. Considering the frequently short or absent discharge conversations, we are uncertain whether all patients were provided with enough time and attention to ensure their understanding of the discharge information and the self‐management activities needed. Although it might be challenging to achieve,^10^, ^38^ we argue that the patients should be provided with information customized to their potential, targeting their individual preferences, even when the time is limited.

Based on our findings, one could argue that the discharge conversation as the main medicines‐related encounter should be re‐evaluated. It has been shown, that same‐day discharge teaching can be ineffective, presumably because patients struggle with anxiety to leave the hospital.^10^, ^39^ Furthermore, many patients struggle to understand and absorb information provided in hospitals, and especially on the day of discharge, many patients may misunderstand the instructions.^2^, ^10^, ^11^, ^34^, ^37^, ^39^ In our study, patients generally experienced a lot of waiting during their hospital stay and this time could have been better utilized. We propose implementing more patient‐centred activities nurturing patient participation during the whole hospital stay, this could reduce the ‘information overload’ in the discharge conversation, as also previously suggested.^40^, ^41^ In addition, it should be further explored how continuity of care could be improved.^7^, ^36^, ^42^, ^43^

A strength of this study is the combination of observations and patient interviews. This makes it possible to both relate to what actually happened, that is mapping the day of discharge, and describe how the patient experienced the discharge.^24^ However, what we remember is not always what happened. An example of this was a patient who, in the interview, talked about a 30‐minute‐long discharge conversation, which when observed lasted for 10 minutes.

The range of observed days in the main study and the broad sample of participants enriched the understanding of the discharge process. Patients were more familiar with the observers when observed for a longer time. We succeeded in recruiting a heterogeneous sample of participants; however, there could be a bias towards more empowered and confident patients, which could impact saturation. Nevertheless, only one patient declined to participate. Saturation was perceived for the main study, which included 15 patients. Some patients were excluded in this specific analysis as they were not interviewed and/or not observed on the day of discharge. Reasons for this were that they withdrew their consent to participate in the interview or that they were moved to another ward before discharge. The nine patients included in the analysis presented in this substudy did not differ from those excluded by any visible character, such as age or sex. As this analysis had a specific aim, full observations and rich interview data, the information power is high although the number of patients is limited.^44^

A limitation with observations can be that the observers filter what they register. However, the observers had continuous discussions about the registrations, and, when possible, observations were audio‐recorded. The Hawthorne (observer) effects were counteracted by a long observation time at the ward (four months), but probably still existed.^45^ In the patient interviews and focus groups with observed HCPs after data collection (unpublished), both groups stated that they had not in any considerable way changed their behaviour because of the observers’ presence. One reason stated for this was that they were used to having student observers present at the ward.

In all research, the sociocultural position of the researchers has an impact on the research process. In this study, the research team consisted of persons with different backgrounds (education, experience of the hospital setting), to involve more perspectives. However, most were female, and all had a Northern European background.

The transferability of the results can be questioned. The study took place in one hospital ward, with its own culture. Observing in different hospitals and/or wards would have increased the observer effect, and hence, the complexity might not have been captured. However, for many of the results, similar outcomes have been shown in other studies (see above).

### 4.2 Conclusion

The study contributes to a broader understanding of the discharge process, how patients experience it, including their role. It is evident that the discharge process is not always tailored to meet the patients’ needs. Both the number of HCPs and encounters challenge the tailoring and alignment of communication. More focus on early patient involvement and communication, in order to better prepare patients in self‐management of their medications, is important for their health outcomes.

## CONFLICT OF INTEREST

The authors declare that they have no competing interests.

## AUTHORS' CONTRIBUTIONS

SER, SKS, MM and LM conceptualized the study and developed the method. YA, HBL and KB contributed to the development of the method. SER, HBL and KB conducted the data collection. SER, SKS, HBL, KB, YA and LM analysed and interpreted the patient data. SER, SKS and LM wrote the original draft. YA, HBL, KB and MM were major contributors to the writing, reviewing and editing. All authors read and approved the final manuscript.

## Data Availability

The data are not publicly available due to privacy or ethical restrictions.
